# Burnout among physicians: Prevalence and predictors of depersonalization, emotional exhaustion and professional unfulfillment among resident doctors in Canada

**DOI:** 10.1192/j.eurpsy.2023.1155

**Published:** 2023-07-19

**Authors:** R. Shalaby, F. Oluwasina, H. El Gindi, E. Eboreime, I. Nwachukwu, M. Hrabok, B. Agyapong, A. Abba-Aji, V. Agyapong

**Affiliations:** 1 Department of Psychiatry, University of Alberta, Edmonton, Canada; 2 Critical Care Medicine, King Abdul-Aziz Hospital, Jeddah, Saudi Arabia; 3 Department of Psychiatry, University of Calgary, Calgary; 4 Department of Psychiatry, University of British Columbia, Vancouver, Canada

## Abstract

**Introduction:**

Burnout in the medical profession has garnered a lot of attention over the recent years. While it is reported across all specialties and all stages of medical education; resident physicians in particular are at high risk for burnout throughout their years of training.

**Objectives:**

This study aimed at evaluating the prevalence and correlates of burnout among resident physicians in Alberta.

**Methods:**

Through a descriptive cross-sectional study design, a self-administered questionnaire was used to gather data from resident physicians at two medical schools in Alberta, Canada. Maslach Burnout Inventory was used as an assessment tool. Chi-squared and multivariate binary logistic regression analyses were used.

**Results:**

Overall burnout prevalence among residents was 58.2%. Working more than 80 hours/week (OR= 16.437; 95% CI: 2.059 – 131.225), being dissatisfied (OR= 22.28; 95% CI: 1.75– 283.278) or being neither satisfied nor dissatisfied with a career in medicine [(OR= 23.81; 95% CI: 4.89 – 115.86) were significantly associated with high depersonalization. Dissatisfaction with efficiency and resources (OR= 10.83; CI: 1.66- 70.32) or being neither satisfied nor dissatisfied with a career in medicine (OR= 5.14; CI: 1.33- 19.94)] were significantly associated with high emotional exhaustion. Working more than 80 hours/week (OR= 5.36; CI: 1.08- 26.42) and feeling that the residency program is somewhat having enough strategies aimed at resident well-being in place (OR= 3.70; CI: 1.10- 12.46) were significantly associated with high work exhaustion and interpersonal disengagement. Young age of the residents (≤ 30 years) (OR= 0.044; CI: 0.004- 0.445) was significantly associated with low professional fulfillment.

**Conclusions:**

Burnout is a serious occupational phenomenon that can degenerate to other conditions or disrupts one’s professional performance. Significant correlates were associated with high rates of burnout. Leaders of medical schools and policy makers need to acknowledge, design, and implement various strategies capable of providing continuous effective mental health support to improve the psychological health of the medical resident across Canada.

**Disclosure of Interest:**

None Declared

